# Binding of Hg(I) and Hg(II) Ions to Amyloid‐Beta (Aβ) Peptide Variants Affect their Structure and Aggregation

**DOI:** 10.1002/cbic.202500252

**Published:** 2025-11-26

**Authors:** Elina Berntsson, Andra Noormägi, Kärt Padari, Jüri Jarvet, Astrid Gräslund, Margus Pooga, Peep Palumaa, Sebastian K. T. S. Wärmländer

**Affiliations:** ^1^ Chemistry Section Stockholm University 10691 Stockholm Sweden; ^2^ Department of Chemistry and Biotechnology Tallinn University of Technology 12618 Tallinn Estonia; ^3^ CellPept Sweden AB Kvarngatan 10B 11847 Stockholm Sweden; ^4^ Institute of Molecular and Cell Biology University of Tartu 50411 Tartu Estonia; ^5^ The National Institute of Chemical Physics and Biophysics 12618 Tallinn Estonia; ^6^ Department of Biophysics and Biochemistry Stockholm University 10691 Stockholm Sweden; ^7^ Institute of Technology University of Tartu 50411 Tartu Estonia

**Keywords:** Alzheimer's disease, amyloid aggregation, heavy metal toxicity, inorganic mercury, metal‐protein binding, neurodegeneration, spectroscopy, transmission electron microscopy

## Abstract

Mercury (Hg) exposure is a possible risk factor for Alzheimer's disease (AD). Some studies reported higher Hg levels in AD patients, but evidence is inconclusive. Mechanisms linking Hg exposure to AD neuropathology remain to be found. The hallmark of AD brains is deposits of insoluble amyloid plaques consisting mainly of aggregated amyloid‐β (Aβ) peptides. Here, we use transmission electron microscopy and biophysical spectroscopy techniques to study in vitro interactions between inorganic Hg and pathologically relevant Aβ(1–40) and Aβ(4–40) variants and the Aβ(1–40)(H6A, H13A, H14A) mutant. For the first time, effects on Aβ aggregation of both Hg(I) and Hg(II) are compared. Hg(II) binds Aβ(1–40) with apparent binding affinity of 28 ± 8 μM, at 20 °C in 20 mM MES buffer, pH 7.3. The N‐terminal His6, His13, and His14 residues are involved in binding coordination. Hg(II) binding induces structural alterations (coil–coil interactions) in Aβ monomers positioned in membrane‐mimicking SDS micelles. Equimolar amounts of either Hg(I) or Hg(II) inhibit normal Aβ fibrillation by directing aggregation towards forming large amorphous aggregates. All these structural rearrangements may be relevant for the harmful Aβ aggregation processes involved in AD brain pathology. Inducing protein misfolding and aggregation might be a general toxic mechanism of mercury.

## Graphical abstract

Mercury (Hg) is a possible risk factor for Alzheimer's disease, where neuronal death is related to amyloid brain plaques containing aggregated amyloid‐β (Aβ) peptides. We show that equimolar Hg(I) or Hg(II) ions inhibit normal Aβ aggregation. Hg(II) binds Aβ(1–40) with an apparent binding affinity 28 ± 8 μM. Inducing protein misfolding and aggregation might be a general toxic mechanism of Hg.

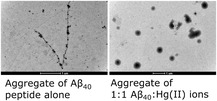

## 1 Introduction

A possible connection between Alzheimer's disease (AD) and environmental mercury (Hg) exposure has been debated for a long time.^[^ ^1^, ^2^, ^3^, ^4^, ^5^, ^6^, ^7^, ^8^, ^9^ ^]^ Hg is a heavy metal with established neurotoxic properties,^[^ ^10^, ^11^ ^]^ and some studies have found higher Hg levels in AD patients.^[^ ^2^, ^8^, ^12^ ^]^ But the evidence is inconclusive, and a number of studies have reported normal Hg levels in both blood and brain of AD patients.^[^ ^8^, ^13^ ^]^ A complicating factor is that AD and mercury intoxication share a common genetic risk factor, namely the APOE‐ε4 gene variant.^[^ ^6^ ^]^ That is, individuals with APOE‐ε4 have an increased probability of developing AD^[^ ^14^ ^]^ and also suffer more severe consequences from Hg exposure.^[^ ^15^, ^16^, ^17^ ^]^ As anthropogenic emissions of mercury continue to increase, mainly due to mining activities and human combustion of coal and oil,^[^ ^18^ ^]^ it becomes important to investigate how Hg might contribute to AD pathology.^[^ ^4^ ^]^

Hg is a neurotoxic and genotoxic heavy metal that induces damage to organs such as the brain and the kidneys.^[^ ^11^, ^15^, ^19^, ^20^ ^]^ Exposure can lead to permanent health problems and even death,^[^ ^16^, ^19^, ^20^ ^]^ with different outcomes depending on dose level and exposure time.^[^ ^21^ ^]^ The molecular mechanisms underlying Hg toxicity are not fully known,^[^ ^4^ ^]^ but include disruption of antioxidant activity^[^ ^22^, ^23^ ^]^ especially in the mitochondria.^[^ ^24^ ^]^ There are also aspects of toxic molecular mimicry,^[^ ^25^ ^]^ as for example complexes between cysteine and methylmercury or inorganic mercury are transported by the amino acid transporter system, probably because these complexes are chemically similar to methionine and/or cystine.^[^ ^26^ ^]^

The different forms of Hg, e.g., metallic, inorganic Hg(I) and Hg(II) species, and organo‐metallic complexes such as methyl‐Hg and ethyl‐Hg, have different chemical properties, toxicity profiles and species distributions.^[^ ^10^, ^11^, ^27^ ^]^ For inorganic Hg(I) and Hg(II) species, the retention time in the human brain is several years, or even decades.^[^ ^28^ ^]^

AD is a progressive, irreversible, and currently incurable chronic neurodegenerative disorder, and also the leading cause of dementia worldwide.^[^ ^29^, ^30^ ^]^ Pathological hallmarks of AD include brain atrophy, with extensive deposits of amyloid plaques and neurofibrillary Tau tangles occurring years before the manifestation of disease symptoms.^[^ ^29^, ^30^ ^]^ The insoluble plaques consist mainly of amyloid‐β (Aβ) peptides^[^ ^31^ ^]^ aggregated into fibrils that display a characteristic cross‐β structure.^[^ ^32^ ^]^ The plaques, which in AD brains contain elevated metal concentrations,^[^ ^33^ ^]^ are the end‐product of an aggregation process^[^ ^34^, ^35^, ^36^ ^]^ that involves formation of extra‐ and intracellular intermediates such as soluble neurotoxic Aβ oligomers.^[^ ^37^ ^]^ As the latter are considered one of the main toxic species in AD brains,^[^ ^38^ ^]^ it becomes imperative to investigate factors that may influence Aβ aggregation and oligomer formation.^[^ ^39^ ^]^

The Aβ peptides are intrinsically disordered in monomeric form and soluble in water.^[^ ^40^ ^]^ The central and C‐terminal Aβ segments are hydrophobic and can interact with membranes or fold into a hairpin conformation that likely is required for aggregation,^[^ ^41^ ^]^ while the anionic N‐terminal segment is hydrophilic and readily interacts with metal ions.^[^ ^40^, ^42^ ^]^ Inorganic Hg(II) has previously been shown to bind to Aβ peptides and interfere with the Aβ aggregation mechanisms.^[^ ^3^, ^43^ ^]^ Specifically, Hg(II) has been reported to increase Aβ_42_ toxicity, promote formation of high‐molecular‐weight soluble aggregates, and reduce the propensity of Aβ_42_ to form harmful ion channels in membranes.^[^ ^44^ ^]^ However, no study has so far investigated Aβ interactions with monovalent Hg(I), even though both Hg(I) and Hg(II) exist in vivo. It is still not clear if the formation and/or activity of toxic Aβ oligomers in AD brains occurs extracellularly, where the oxidizing environment yields Hg(II), or/and intracellularly, where the reducing environment yields Hg(I).

In this study, we use transmission electron microscopy (TEM) imaging together with fluorescence, CD, and nuclear magnetic resonance (NMR) spectroscopy to study in vitro interactions between Aβ peptides and inorganic Hg ions. The focus is on Hg binding properties and Hg binding effects on Aβ structure and aggregation. Both Hg(I) and Hg(II) are investigated and compared for the first time. The studied Aβ peptides are the pathologically relevant Aβ(1–40) and Aβ(4–40) variants, together with the Aβ(1–40)(H6A, H13A, H14A) mutant (i.e., Aβ_NoHis_) that is used to investigate the importance of the Aβ His residues for Hg binding.

## 2 Results

### 2.1 TEM Imaging

**Figure 1:**
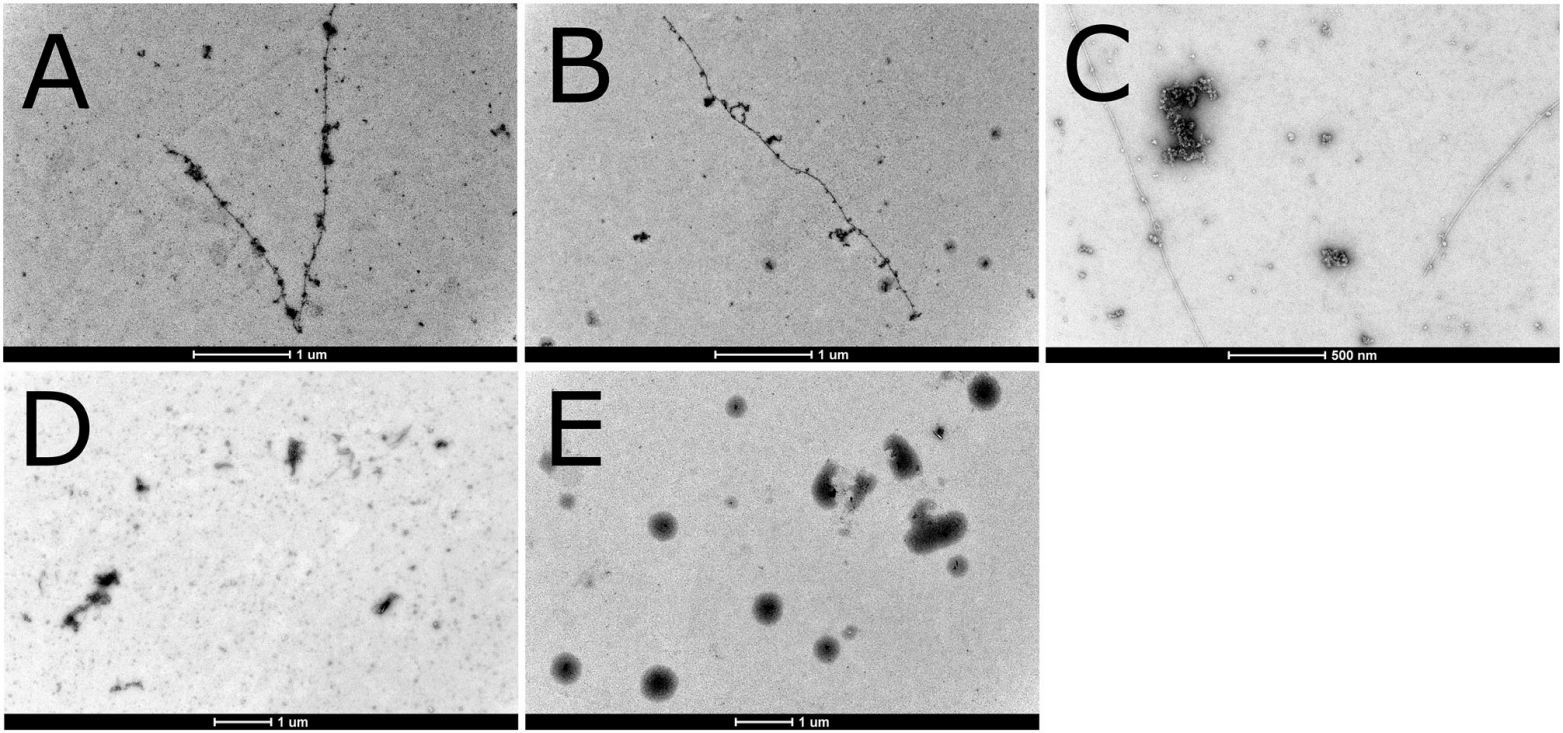
Negative staining TEM images for aggregates of 10 μM Aβ_40_ peptide in 20 mM MES buffer, pH 7.3, incubated for 20 h on a thermo shaker at 37 °C and 300 rpm, together with different amounts of Hg(II). A) 0 μM Hg(II). B) 0.5 μM Hg(II). C) 1.0 μM Hg(II). D) 3.0 μM Hg(II). E) 10 μM Hg(II). The white scale bars are 1 μm. Images by K.P.

**Figure 2:**
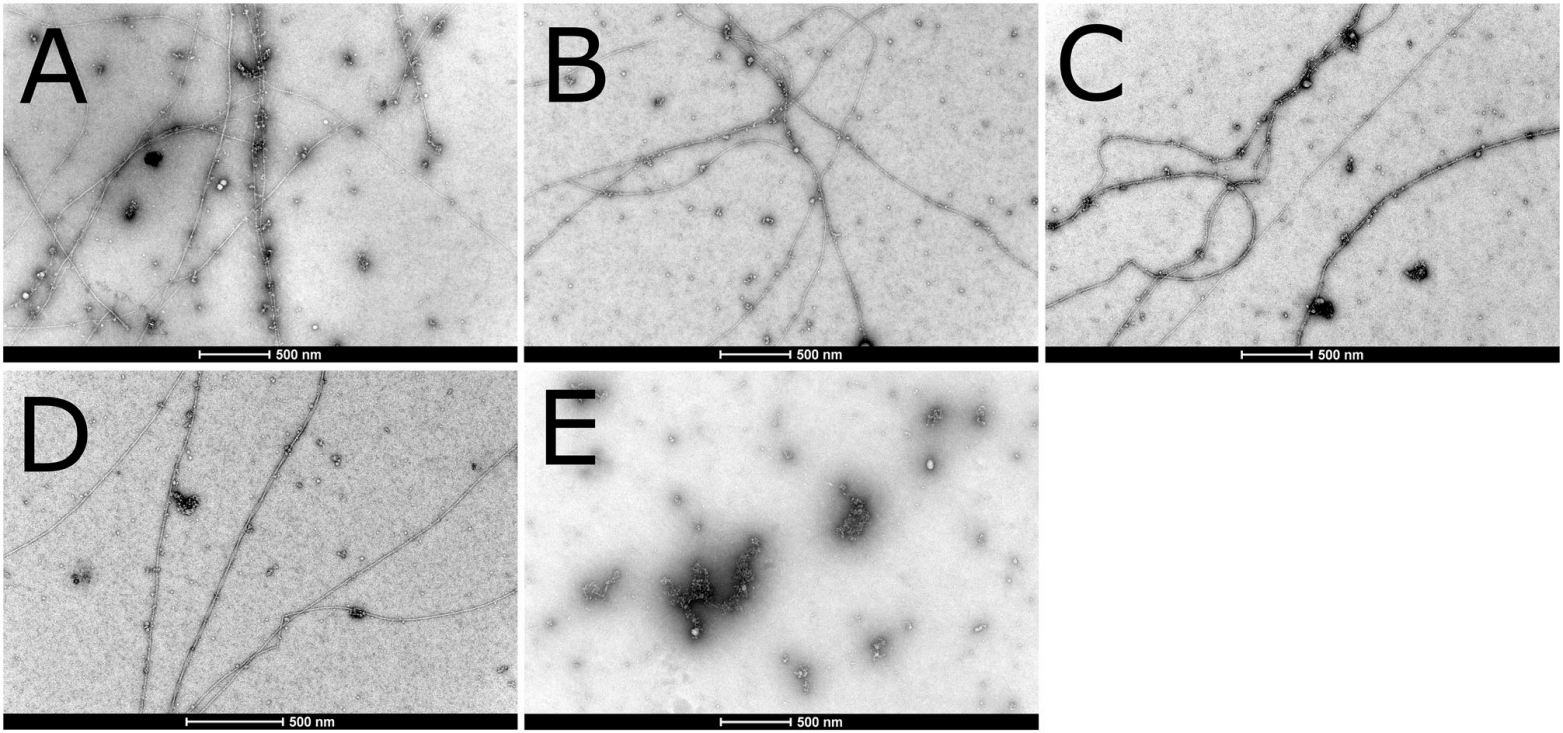
Negative staining TEM images for aggregates of 10 μM Aβ_40_ peptide in 1 mM TCEP and 20 mM MES buffer, pH 7.3, incubated for 20 h on a thermo shaker at 37 °C and 300 rpm, together with different amounts of Hg(I). A) 0 μM Hg(I). B) 0.5 μM Hg(I). C) 1.0 μM Hg(I). D) 3.0 μM Hg(I). E) 10 μM Hg(I). The white scale bars are 500 nm. Images by K.P.

TEM images were recorded to study the morphologies of aggregates of 10 μM Aβ_40_ peptide, incubated for 20 h together with different concentrations of Hg(NO_3_)_2_. The incubations were carried out either in MES buffer only (**Figure** **1**), or together with 1 mM of the reducing agent TCEP (**Figure** **2**).

Under standard oxidizing conditions, i.e., without TCEP present, the aggregated control sample of Aβ_40_ peptide without added mercury displays slender fibrils that are several microns long, together with smaller aggregate particles that might be protofibrils (Figure 1A). This is in line with previous in vitro studies of the size and shape of self‐aggregated Aβ_40_ fibrils,^[^ ^45^ ^]^ which appear to grow out from globular protofibrillar assemblies.^[^ ^46^ ^]^ A similar fibril morphology is observed for the Aβ_40_ samples incubated with 0.5 μM Hg(II) ions (Figure 1B). The Aβ_40_ sample with 1 μM Hg(II) ions is somewhat different, displaying a mixture of slender fibrils, small aggregate particles, and larger amorphous aggregates (Figure 1C). The Aβ_40_ samples with 3 μM and 10 μM Hg(II) contain no fibrils, but only small and large aggregate particles (Figure 1D,E). The sample with 10 μM Hg(II) is dominated by large aggregated particles, roughly half a micron across (Figure 1E). This shows that Hg(II) ions promote formation of large amorphous aggregates rather than slender fibrils. The effect is clearly concentration‐dependent, and the formation of long fibrils is completely inhibited already at substoichiometric mercury concentrations, i.e., with 3 μM Hg(NO_3_)_2_ to 10 μM Aβ_40_ peptide (Figure 1D).

For the samples incubated under reducing conditions, i.e., with 1 mM TCEP present, the Aβ_40_ control sample without added mercury again displays numerous amyloid fibrils, several microns long, together with small aggregate particles (Figure 2A). The Aβ_40_ samples incubated together with 0.5, 1, and 3 μM Hg(I) display similar combinations of amyloid fibrils and small amorphous aggregates (Figure 2B–D). At 10 μM added Hg(I) the Aβ_40_ peptide no longer forms slender fibrils, but rather amorphous aggregates in various sizes (Figure 2E). This shows that Hg(I) has a similar, albeit weaker, concentration‐dependent effect on Aβ_40_ aggregation as Hg(II). For example, complete inhibition of Aβ_40_ fibrillation is achieved by Hg(I) at stoichiometric concentrations (1:1 Hg(I):Aβ_40_ ratio; Figure 2E), but by Hg(II) already at substoichiometric concentrations (3:10 Hg(II):Aβ_40_ ratio; Figure 1D).

### 2.2 Fluorescence Measurements of Hg(II) Binding Affinity

**Figure 3:**
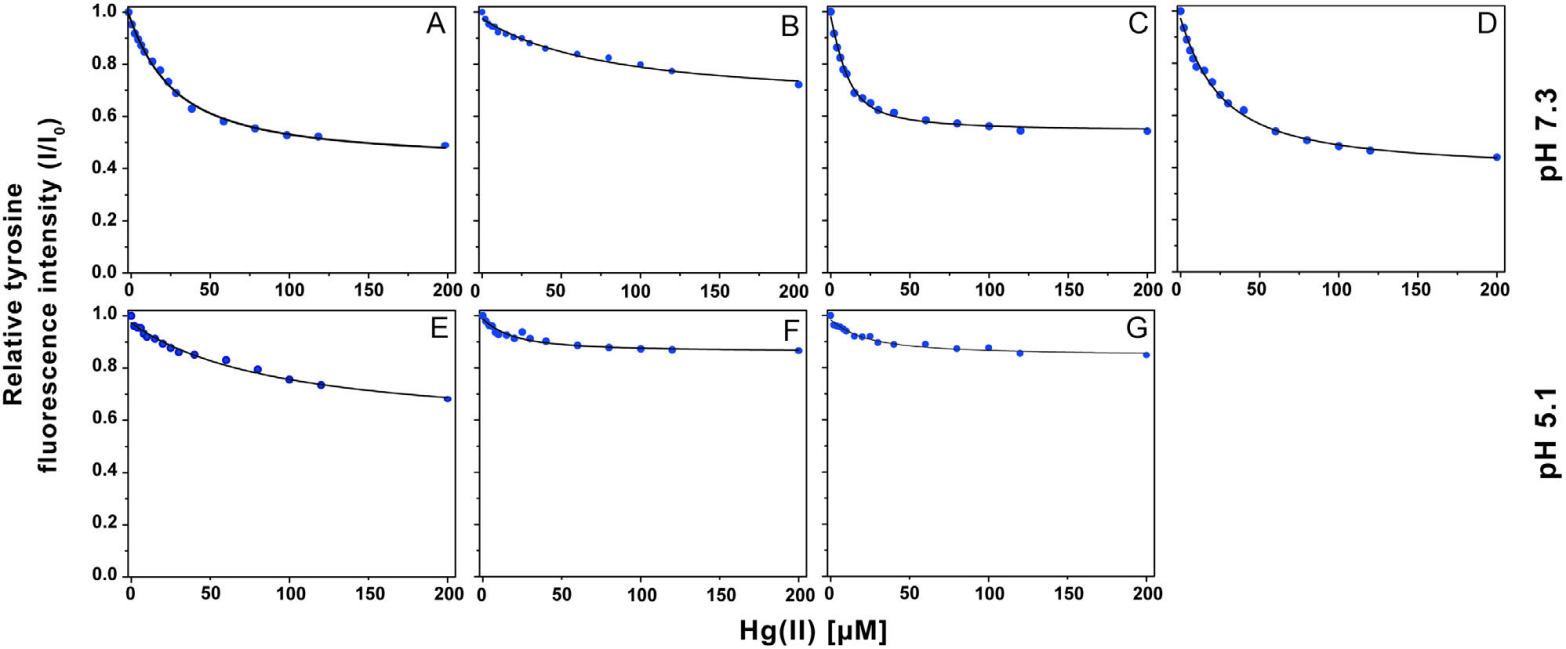
Changes in intrinsic fluorescence of Tyr10 in Aβ peptide variants upon addition of Hg(II). All measurements were carried out with 20 μM Aβ peptide and 20 mM MES buffer at 20 °C. Top row: pH 7.3. Bottom row: pH 5.1. A,E) Aβ_40_. B,F) Aβ_NoHis_. C,G) Aβ_4–40_. D) Aβ_40_ with 50 mM SDS.

Hg ions have previously been shown to be able to quench the intrinsic fluorescence of Tyr residues,^[^ ^47^ ^]^ similar to, e.g., Ag(I), Cu(II), and UO_2_(II) ions.^[^ ^48^, ^49^, ^50^, ^51^, ^52^ ^]^ The effect of added Hg(II) on the fluorescence of Tyr10, the only natural fluorophore in Aβ peptides, was therefore used to evaluate binding affinities for Hg(II)·Aβ complexes in different conditions (**Figure** **3**), using three replicates for each Aβ variant and/or condition.

For titrations with Hg(II) at pH 7.3, the fluorescence intensity curves can be fitted to Equation (1) with good accuracy (Figure 3). This shows that the binding interactions are dominated by a single binding site. As seen in **Table** **1**, Hg(II) displays the strongest binding to the Aβ_4–40_ variant (4 ± 2 μM), weakest binding to Aβ_NoHis_ (60 ± 18 μM), and intermediate binding affinity to wild‐type Aβ_40_ (28 ± 8 μM). The apparent dissociation constant for the Hg(II)·Aβ_40_ complex in the presence of sodium dodecyl sulfate (SDS) micelles is 20 ± 9 μM, which is very similar to the 28 ± 8 μM value observed in buffer only (Figure 3, Table 1). Although Aβ peptides are known to interact with SDS micelles,^[^ ^53^, ^54^ ^]^ which are a very simple membrane model, these interactions apparently have no significant effect on the Hg(II) binding affinity.

**Table 1:** Apparent dissociation constants ($K_{D}^{\text{App}}$) in μM for Aβ·Hg(II) complexes, measured at different pH and with different Aβ peptide variants in 20 mM MES buffer at 20 °C.

	Sample 1	Sample 2	Sample 3	Mean $K_{D}^{\text{App}}$ [μM]a)
Aβ_40_ pH 7.3	38.8	22.9	23.6	28 ± 8
Aβ_40_ pH 5.1	14.6	87.2	32.9	45 ± 31
Aβ_4–40_ pH 7.3	4.9	2.5	3.0	4 ± 2
Aβ_4–40_ pH 5.1	16.9	10.3	6.4	8 ± 5
Aβ_NoHis_ pH 7.3	70.3	74.8	35.2	60 ± 18
Aβ_NoHis_ pH 5.1	5.8	13.2	3.5	11 ± 5
Aβ_40_ pH 7.3 + 50 mM SDS	30.1	19.8	10.3	20 ± 9

a) The $K_{D}^{\text{App}}$ values were obtained by fitting Equation (1) to the binding curves shown in Figure 3. Three separate samples were prepared and measured for each condition, labeled 1, 2, and 3 in the table. Mean values and standard deviations for the three samples are shown in the rightmost column.

At pH 5.1, the overall fluorescence quenching effect is smaller, possibly suggesting generally weaker metal binding at acidic pH (Figure 3). Due to this smaller effect, the $K_{D}^{\text{App}}$ values derived at pH 5.1 have larger errors than those at pH 7.3 (Table 1). The Hg(II) affinity values at pH 5.1 are 45 ± 31 μM for Aβ_40_, 8 ± 5 μM for Aβ_4–40_, and 11 ± 5 μM for Aβ_NoHis_ (Table 1). Both Aβ_40_ and Aβ_4–40_ display weaker binding to Hg(II) at pH 5.1 than at pH 7.3. However, for neither peptide the difference is statistically significant, mainly due to the larger errors at pH 5.1 (Figure 3; Table 1). For Aβ_NoHis_, the Hg(II) binding is instead somewhat stronger at lower pH (Table 1).

The most reasonable explanation for weaker Hg(II) binding to Aβ_40_ and Aβ_4–40_ at acidic pH is protonation of His residues in Aβ, as this would disfavor binding of cations. This implies that His6, His13 and/or His14 are involved as binding ligands, which is supported by Aβ_NoHis_ displaying weaker Hg(II) binding than Aβ_40_ and Aβ_4–40_ at neutral pH (Table 1). Also, as there are no His residues that can be protonated in Aβ_NoHis_, this peptide variant does not show weaker Hg(II) binding at acidic pH (Table 1).

Binding titrations were also carried out with Hg(I), i.e., under reducing conditions obtained by addition of 1 mM TCEP to the samples. These titrations were not successful, as they did not produce meaningful binding curves (data not shown).

### 2.3 NMR Spectroscopy

**Figure 4:**
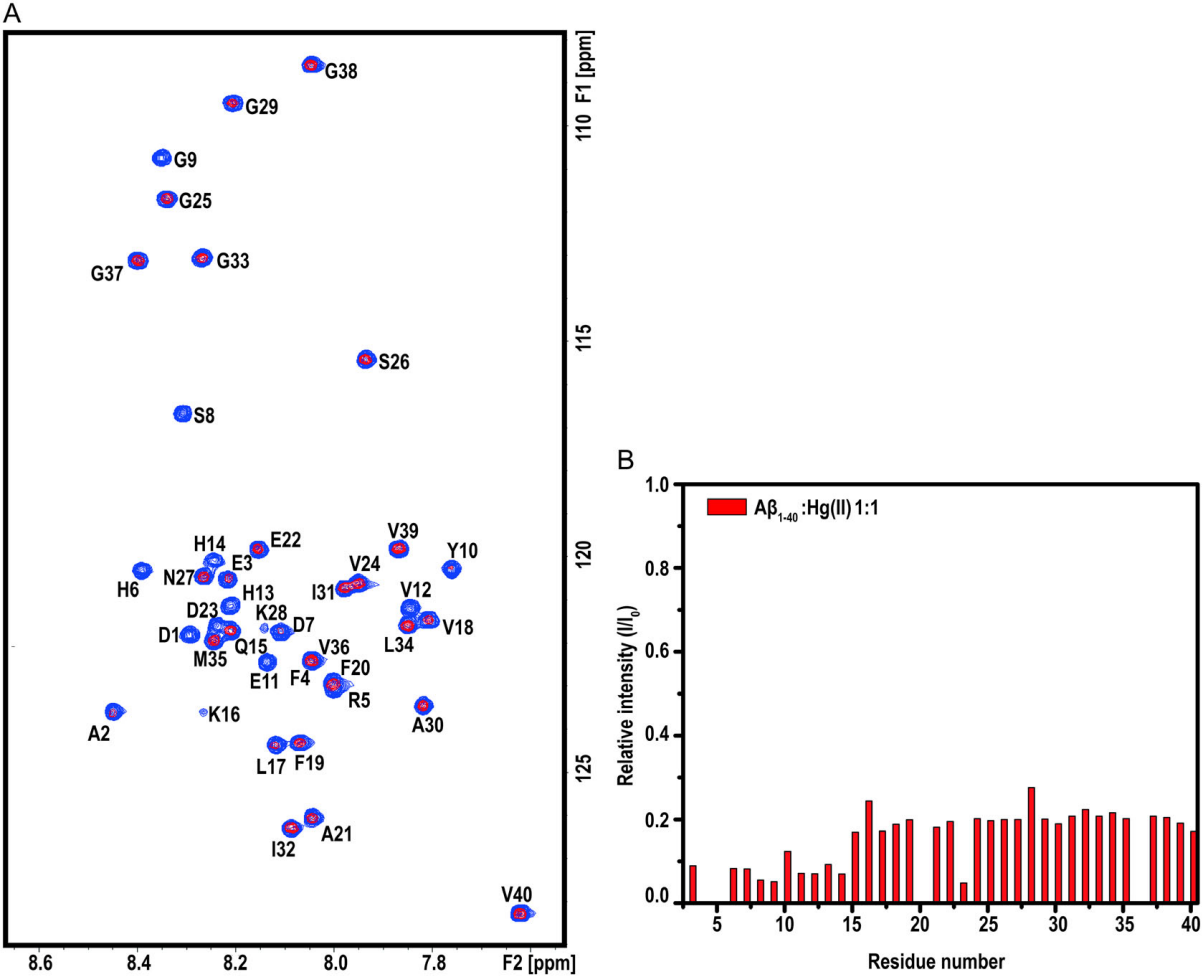
A) NMR spectra (2D ^1^H,^15^N‐HSQC) of 92 μM monomeric ^15^N‐labeled Aβ_40_ peptide in 20 mM MES buffer, pH 5.1, recorded at 5 °C before (blue crosspeaks) and after (red crosspeaks) addition of 92 μM Hg(NO_3_)_2_. B) Relative crosspeak intensities for individual Aβ_40_ residues, i.e., I/I_0_, showing the effect of added Hg(II) at a 1:1 ratio.

High‐resolution NMR experiments were performed to investigate possible residue‐specific molecular interactions between Hg(II) and monomeric Aβ_40_ peptide (**Figure** **4**). The experiments were conducted at pH 5.1, where histidine residues are protonated. Figure 4A shows 2D ^1^H,^15^N‐HSQC spectra for the amide crosspeak region of the Aβ_40_ peptide, recorded before and after addition of Hg(II) in a 1:1 Hg(II):Aβ_40_ ratio. Although there is a general loss of signal intensity for all residues after added Hg(NO_3_)_2_, the signal loss is most pronounced in the N‐terminal segment (Figure 4B). This shows that there are specific binding interactions between Hg(II) and certain N‐terminal residues. Because Hg(II) is diamagnetic with a 5*d* ^10^ electronic configuration, the increased loss of crosspeak intensity for these amino acids is not caused by paramagnetic effects. Instead, it likely results from intermediate chemical exchange on the NMR time‐scale,^[^ ^55^ ^]^ between the Hg(II)·Aβ_40_ complex and free Aβ_40_ peptide. This could be similar to the effects previously observed when Aβ peptide interacts with diamagnetic Zn(II) ions.^[^ ^56^, ^57^, ^58^ ^]^

### 2.4 CD Spectroscopy

**Figure 5:**
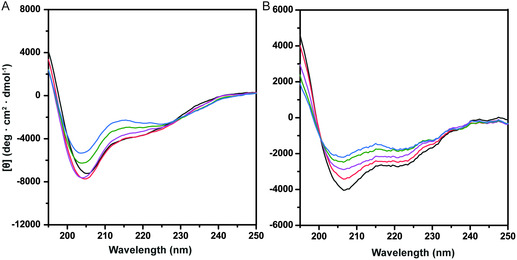
A) CD spectra of 10 μM Aβ_40_ peptide and B) 10 μM Aβ_4–40_ peptide in 20 mM sodium phosphate buffer, pH 7.3, titrated with Hg(NO_3_)_2_ at 20 °C in the presence of SDS (50 mM) micelles. Black: Aβ peptide only. Red: 16 μM Hg(II). Purple: 56 μM Hg(II). Green: 156 μM Hg(II). Blue: 256 μM Hg(II).

CD spectroscopy was used to monitor possible effects of Hg(II) on the secondary structure of monomeric Aβ_40_ and Aβ_4–40_ peptides, investigated in a membrane‐mimicking environment consisting of SDS micelles. In this environment and without added Hg(NO_3_)_2_, both peptides display CD spectra characteristic for α‐helix structure (**Figure** **5**), i.e., with typical minima around 222 and 208 nm.^[^ ^59^ ^]^ The spectra of the two peptides are however not identical, as they differ in the ratios between the CD intensities at 208 and 222 nm (**Table** **2**). This shows that the two peptide variants have slightly different α‐helix conformations in micelles, which is consistent with earlier studies showing Aβ_4–40_ in SDS micelles to have a higher 222/208 ratio than Aβ_40_.^[^ ^60^ ^]^ Furthermore, the overall α‐helix CD signal is stronger for Aβ_40_ (–6300 at 208 nm) than for Aβ_4–40_ (–3900 at 208 nm), which is in line with earlier measurements of these two peptides.^[^ ^60^ ^]^

**Table 2:** CD signal intensities at 208 and 222 nm for the Aβ_40_ and Aβ_4–40_ peptide variants, as a function of added Hg(II).

	Wavelength [nm]	0 [μM] Hg(II)	16 [μM] Hg(II)	56 [μM] Hg(II)	156 [μM] Hg(II)	256 [μM] Hg(II)a)
Aβ_40_	208	–6332	–6510	–6123	–4965	–4055
	222	–3406	–3447	–3093	–2865	–2522
	222/208 ratio	0.538	0.538	0.529	0.505	0.577
Aβ_4–40_	208	–3974	–3351	–2827	–2357	–2100
	222	–2679	–2446	–2169	–1851	–1772
	222/208 ratio	0.67	0.73	0.77	0.78	0.84

a) The values are derived from the CD spectra shown in Figure 5.

Addition of Hg(II) induces similar concentration‐dependent structural transitions in both peptide variants (Figure 5). For the Aβ_4–40_ peptide, the start and end spectra have similar characteristic minima around 208 and 222 nm, and there is an isodichroic point around 200 nm (Figure 5B). This shows that the transition is from one well‐defined α‐helical conformation to another. The main difference between the start and end spectra is the 222/208 ratio, which increases from 0.67 without Hg(II) to 0.84 with 256 μM added Hg(II) (Table 2). Such spectral changes are known to correspond to changes in helix supercoiling, i.e., when two or more α‐helices form coiled coils via hydrophobic interactions.^[^ ^61^, ^62^, ^63^ ^]^ In such structures, a 222/208 ratio close to 1 reflects large amounts of superhelicity.^[^ ^64^ ^]^

For the Aβ_40_ peptide, addition of Hg(II) induces a structural transition (Figure 5A) that is similar but not identical to the one observed for the Aβ_4–40_ peptide (Figure 5B). That is, for Aβ_40_, there is no clear isodichroic point (Figure 5A), and the 222/208 ratios remain around 0.5 during the titration (Table 2). It is therefore less clear how bound Hg(II) ions modulate the α‐helical structure of wildtype Aβ_40_ peptide.

## 3 Discussion

Harmful metal exposure has for a long time been suspected to contribute to neurodegenerative diseases.^[^ ^1^, ^8^, ^9^, ^65^, ^66^, ^67^, ^68^, ^69^ ^]^ Thus, both endogenous metals such as Cu, Fe, and Zn and numerous exogenous metals have been investigated in relation to AD, either by measuring metal concentrations in blood and brains of AD patients,^[^ ^8^, ^12^, ^70^ ^]^ or by in vitro studies of metal ion interactions with disease‐related molecules such as Aβ and tau.^[^ ^40^, ^42^, ^71^, ^72^, ^73^, ^74^, ^75^ ^]^

Our TEM images show that both Hg(I) and Hg(II) affect Aβ_40_ peptide aggregation, by inhibiting fibril formation and instead promoting formation of large amorphous clump‐like aggregates (Figure 1 and 2). The effect is somewhat stronger for Hg(II), which completely inhibits fibril formation already at substoichiometric concentrations (Figure 1D), while Hg(I) inhibits fibrillation at stoichiometric amounts (Figure 2E). These results support earlier studies on the effect of Hg(II) on Aβ fibrillation.^[^ ^3^ ^]^ Similar effects on Aβ aggregation have also been observed for ions of the heavy metals Ag, Cd, Pb, and U.^[^ ^45^, ^51^, ^52^, ^57^ ^]^ As Hg has been found to induce harmful aggregation of other proteins such as *γ*‐crystallins^[^ ^76^ ^]^ and ovalbumin,^[^ ^77^ ^]^ it is possible that one toxic mechanism of mercury, and perhaps of heavy metals in general, could be to induce misfolding and aggregation of proteins and peptides.^[^ ^78^, ^79^ ^]^

It therefore appears very likely that the aggregation‐modulating properties of Hg(I) and Hg(II) would affect the structure and toxicity of Aβ oligomers, which are considered a main toxic species in AD neuropathology.^[^ ^37^, ^38^ ^]^ However, due to the intrinsic Hg toxicity, it is difficult to compare the toxicities of Aβ aggregates formed in the presence or absence of Hg. It has still not been clarified if harmful Aβ aggregation occurs intracellularly or extracellularly in AD brains. Because the Hg(II) form exists in oxidizing environments, i.e., extracellularly, and the Hg(I) form exists in reducing environments, i.e., intracellularly, the TEM images in Figure 1 and 2 indicate that Hg ions may interfere with Aβ aggregation both inside and outside neural cells.

The fluorescence quenching experiments show that Hg(II) binds Aβ_40_ peptide with apparent dissociation constants in the micromolar range at 20 °C in 20 mM MES buffer (Figure 1; Table 1). At pH 7.3, Hg(II) binds wildtype Aβ_40_ peptide with an apparent dissociation constant of 28 ± 8 μM. Because the values in Table 1 have not been corrected for buffer effects, the true affinities should be somewhat stronger.^[^ ^50^ ^]^ The Hg(II) binding affinity is similar to Aβ_40_ binding to Ag(I), Ni(II), and Zn(II),^[^ ^52^, ^56^, ^60^ ^]^ stronger than binding to Li(I), Mn(II), and uranyl ions,^[^ ^51^, ^80^, ^81^ ^]^ but weaker than binding to Cu(II).^[^ ^48^, ^50^ ^]^

The somewhat stronger binding of Hg(II) to Aβ_4–40_ than to wildtype Aβ_40_ (4 ± 2 vs. 28 ± 8 μM; Table 1) suggests that Hg(II) could display stronger binding to ATCUN (amino‐terminal Cu,Ni‐binding) motifs. These motifs consist of an N‐terminal tripeptide with a His residue at position three, i.e., X‐Z‐His, and are known to strongly bind metal ions such as Cu(II) and Ni(II).^[^ ^82^, ^83^, ^84^ ^]^ Truncating the first three residues in Aβ_40_ produces an ATCUN motif, as His6 then becomes residue number three. Because Aβ_4–40_ and other N‐terminally truncated peptide variants are abundant in both healthy and AD brains,^[^ ^82^, ^85^ ^]^ it has been argued that the observed stronger Cu(II) binding to the Aβ_4–40_ peptide could be of importance in AD progression.^[^ ^82^, ^84^, ^86^ ^]^ Cu(II) and Ni(II) have the electron configurations [Ar]3*d* ^9^ and [Ar]3*d* ^8^, respectively, and usually prefer square planar or tetrahedral binding configurations with a coordination number of four.^[^ ^87^ ^]^ Hg(II) has the electron configuration [Xe]4*f* ^14^5*d* ^10^, and can adopt a wide range of coordination geometries, such as octahedral, T‐shaped, square planar, linear, and tetrahedral.^[^ ^88^, ^89^, ^90^ ^]^ Thus, it is conceivable that Hg(II) might benefit from the advantageous binding properties of ATCUN binding sites, just like Cu(II) and Ni(II).

The stronger binding to the ATCUN motif in Aβ_4–40_ implies that His6 is involved as a binding ligand for Hg(II). Hg ions often bind to proteins via thiol or selenol groups,^[^ ^7^, ^23^, ^91^, ^92^, ^93^ ^]^ which are not present in Aβ peptides. Instead, earlier studies have suggested that the three N‐terminal His residues, i.e., His6, His13 and His4, could be involved in coordinating Hg(II) to Aβ.^[^ ^3^ ^]^ This is supported by the weaker Hg(II) binding affinity to the Aβ_NoHis_ mutant, i.e. 60 ± 18 μM at pH 7.3, compared to the 28 ± 8 μM observed for wildtype Aβ_40_ (Table 1). Additional evidence is provided by the weaker binding of Hg(II) to both Aβ_40_ and Aβ_4–40_ at acidic pH, compared to the affinities at neutral pH (Table 1). The most reasonable explanation for these results is protonation of the three Aβ His residues, which have pKa values around 6.^[^ ^94^ ^]^ Protonated binding ligands are of course less effective at binding cations. However, our NMR results show that even at acidic pH, Hg(II) still displays residue‐specific binding to the N‐terminal Aβ region up to the His14 residue (Figure 4B). Earlier work has demonstrated that Cu(II) binding to Aβ peptides occurs via multiple binding configurations, which involve different combinations of His6, His13, His14, and anionic residues such as Asp1 and Glu11.^[^ ^75^ ^]^ It is quite possible that a similar ensamble of different binding configurations exists also for Hg(II) binding to Aβ_40_ peptide, especially given the propensity of Hg(II) to adopt many different coordination geometries.^[^ ^88^, ^89^, ^90^ ^]^

No binding affinities, nor proper binding curves, were obtained for Hg(I) binding to Aβ. The reason is unclear but likely related to the complexity of the system used for the Hg(I) measurements. The Hg(II) ions were here reduced to Hg(I) by the common reducing molecule TCEP (tris(2‐carboxyethyl)phosphine), which can form complexes with metal ions.^[^ ^95^, ^96^ ^]^ When reduced to Hg(I), diatomic Hg_2_^2+^ ions are often formed.^[^ ^90^, ^97^ ^]^ It has previously been shown that Aβ peptides generate oxygen radicals,^[^ ^98^, ^99^ ^]^ especially in complex with redox‐active Cu or Fe ions,^[^ ^100^, ^101^ ^]^ and thus likely also with redox‐active Hg ions. These redox reactions cause molecular damage that probably is an important factor in AD progression,^[^ ^42^, ^102^ ^]^ and might oxidize Hg(I) bound to Aβ peptides to the Hg(II) state. It is therefore unclear which form of Hg that interacts with the Aβ peptides when TCEP is present in the solution. Future studies will hopefully shed more light on how Hg(I) interacts with proteins and peptides under reducing conditions that correspond to an intracellular environment.

A recent study reported apparent binding affinities around 5 μM for Hg(II)·ApoE complexes,^[^ ^47^ ^]^ which is in the same order as the Hg(II)·Aβ affinities reported in this work (Table 1). Similar to Aβ peptides, the ApoE binding residues for Hg ions appear to be histidines. Hg(I) appeared to bind weaker than Hg(II) to the ApoE proteins,^[^ ^47^ ^]^ and a similar relationship might exist for Hg(I) and Hg(II) binding to Aβ peptides, although this should be further investigated. Interestingly, the ApoE protein study demonstrated almost identical Hg(II) binding affinities to the three ApoE protein variants ApoE2, ApoE3, and ApoE4.^[^ ^47^ ^]^ This result refuted the idea that the missing cysteines in ApoE4 would make it a worse Hg transporter than ApoE2 and ApoE3, which previously was considered the most likely explanation for ApoE4 being a risk factor in Hg exposure.^[^ ^5^, ^6^, ^15^, ^17^ ^]^ Now, other explanations need to be considered and investigated.

The binding affinity of the Hg(II)·Aβ_40_ complex is not much changed when micelles of 50 mM SDS are added to the sample, i.e., from 28 ± 8 to 20 ± 9 μM (Table 1). The 50 mM of SDS molecules corresponds to slightly less than 1 mM of micelles, which is much higher than 10 μM Aβ peptide. Consequently, there is arguably no more than one Aβ peptide in each micelle. It is well established that the central and C‐terminal hydrophobic Aβ segments insert themselves into SDS micelles, where they form two separate α‐helices.^[^ ^53^, ^103^ ^]^ The hydrophilic N‐terminal Aβ segment is hanging outside the membrane surface, where it is free to bind e.g. metal ions. Thus, the results reported here are in line with previous studies showing that Aβ N‐terminal metal binding is not much affected when the peptide is bound to membranes or membrane mimetica.^[^ ^48^ ^]^ The current results also suggest that Hg(II) binding should be very similar for Aβ_40_ and the slightly longer Aβ_42_ peptide, as the two additional hydrophobic C‐terminal residues in Aβ_42_ should not affect the N‐terminal metal binding properties.

The CD results show that addition of Hg(II) induces concentration‐dependent changes in the α‐helical structure of Aβ peptides positioned in SDS micelles (Figure 5). For Aβ_4–40_, the structural transition involves Hg(II)‐induced formation of coil–coil interactions (Figure 5B), similar to previously observed effects of e.g., Cu(II) and Ni(II) ions.^[^ ^49^, ^60^ ^]^ For Aβ monomers in SDS, the coil–coil interactions most likely result from the two α‐helical segments 15–25 and 29–35 interacting with each other.^[^ ^53^ ^]^ For Aβ_40_, the nature of the structural changes is less clear and may involve other secondary structures than α‐helix (Figure 5A). The observed structural rearrangements of Aβ peptides in a membrane‐mimicking environment could be biologically relevant, as membrane disruption is one likely toxic mechanism of Aβ oligomers.^[^ ^42^ ^]^ Structural alterations are also a likely explanation for the previously reported observation that binding of Hg(II) reduces the propensity of Aβ_42_ to form ion channels in membranes.^[^ ^44^ ^]^

In humans and other organisms, Hg ions are detoxified and excreted from the body via binding to proteins such as metallotheoneins and glutathione.^[^ ^104^, ^105^, ^106^ ^]^ These proteins strongly bind Hg ions mainly via thiol groups in e.g., cysteine residues,^[^ ^7^, ^23^, ^92^, ^107^ ^]^ which display binding affinities for Hg(II) in the nanomolar range.^[^ ^93^ ^]^ Our results show that monomeric Aβ peptides bind Hg(II) with apparent affinities in the low micromolar region (Table 1), i.e., roughly in the same affinity range as Aβ binding to Cu(II), Ni(II), and Zn(II) ions.^[^ ^48^, ^56^, ^60^ ^]^ This indicates that Aβ monomers will not be able to compete with Hg(II) binding to glutathione and metallotheoneins in vivo, especially as both Hg ions and Aβ peptides typically are present only in nanomolar concentrations in human fluids.^[^ ^108^, ^109^, ^110^ ^]^

However, it has been shown that metals such as Cu, Fe, and Zn,^[^ ^33^, ^111^ ^]^ and perhaps also Hg,^[^ ^112^ ^]^ accumulate in the amyloid plaques in AD brains. Thus, it appears that these plaques, which consist mainly of aggregated Aβ peptides,^[^ ^31^ ^]^ bind metal ions more strongly than Aβ monomers do. We therefore speculate that Hg(II) might display biologically relevant in vivo interactions with aggregated Aβ species in brain plaques. Such interactions would probably be harmful. For example, it has been proposed that the observed crosslinking of aggregated Aβ peptides in AD plaques could be caused by oxygen radicals created by redox‐active metal ions such as Cu(II) and Fe(II),^[^ ^113^ ^]^ and possibly also by Hg(II).

## 4 Conclusions

Hg(II) binds to the N‐terminal segment of biologically relevant Aβ peptide variants, where the three N‐terminal His residues are involved as binding ligands. In 20 mM MES buffer and at 20 °C, the Hg(II) binding affinity is in the low μM range. Hg(II) binding induces structural alterations in Aβ monomers positioned in membrane‐mimicking SDS micelles. Equimolar amounts of either Hg(I) or Hg(II) inhibit normal Aβ fibrillation by instead directing the aggregation process towards formation of large amorphous aggregates. As Aβ aggregation is considered a major toxic mechanism in AD progression, the observed Hg‐induced structural rearrangements likely affect AD neuropathology. The capacity to induce misfolding and aggregation of proteins and peptides might be a general toxic mechanism of mercury and other heavy metals.

## 5 Experimental Section

5.1

5.1.1

##### Materials

Recombinantly produced wild‐type (wt) Aβ(1–40) peptide, abbreviated as Aβ_40_, with the primary sequence DAEFR_5_HDSGY_10_EVHHQ_15_KLVFF_20_AEDVG_25_SNKGA_30_IIGLM_35_VGGVV_40_, were purchased as lyophilized powder from AlexoTech AB (Umeå, Sweden). Recombinantly produced truncated Aβ(4–40) peptide, abbreviated as Aβ_4–40_, Aβ(1–40)(H6A, H13A, H14A) mutant peptide, abbreviated as Aβ_NoHis_, and uniformly ^15^N‐labeled Aβ_40_ peptide were also purchased from AlexoTech AB. All Aβ peptide variants were stored at −80 °C until use, when they were dissolved to monomeric form in 10 mM NaOH. The fresh solutions were sonicated for 5 min in an ice‐bath to dissolve possible pre‐formed aggregates. Then, phosphate buffer or 2‐(N‐morpholino)ethanesulfonic acid hydrate (MES) buffer was added. All preparation steps were performed on ice. The peptide concentrations were first estimated from the weight of the dry powder and then more accurately determined by measurements of the solutions with a NanoDrop instrument. The reducing agent TCEP (tris(2‐carboxyethyl)phosphine) was added to some samples, in order to reduce Hg(II) to Hg(I).

##### TEM Imaging

Negative staining TEM images were recorded for 10 μM Aβ_40_ peptide in 20 mM MES buffer, pH 7.3, that had been incubated for 20 h on a thermo shaker operating at 37 °C and 300 rpm, together with either 0, 0.5, 1, 3, or 10 μM of Hg(NO_3_)_2_. Then, 5 μL of each incubated sample were put on copper grids of 200 μm mesh size. The grids were covered with Pioloform film upon which a carbon layer had first been deposited and then glow‐discharged using a Leica EM ACE600 carbon coater (Leica Microsystems, Germany). The Aβ_40_ samples were absorbed to the grids for 5 min, rinsed with Milli‐Q water two times, and then stained for 2 min with 2% aqueous solution of uranyl acetate as a contrast agent for the images. The excess stain was removed with filter paper, and then the samples were left to air‐dry. A digital Orius SC1000 camera was used to record TEM images in a FEI Tecnai G2 Spirit electron microscope (FEI, The Netherlands) operating at 120 kV accelerating voltage.

To investigate the effects of both monovalent Hg(I) and divalent Hg(II) on Aβ aggregation, all samples were prepared and incubated in both oxidizing and reducing conditions, respectively. The reducing condition, roughly corresponding to the chemical environment inside the cell cytosol, was obtained by adding 1 mM of the reducing agent TCEP to the samples. This yields monovalent Hg(I), which typically exists as diatomic Hg_2_^2+^ ions.^[^ ^90^, ^97^ ^]^ Standard oxidizing conditions yield the Hg(II) form, which typically exists as free monoatomic Hg^2+^ ions.

##### Fluorescence Spectroscopy

Fluorescence measurements were carried out with a Jobin Yvon Horiba Fluorolog 3 fluorescence spectrometer (Longjumeau, France). Emission intensities at 306 nm (excitation 276 nm) were recorded at room temperature (20 °C) for samples of 20 μM Aβ peptide (Aβ_40_, Aβ_NoHis_, or Aβ_4–40_) in 20 mM MES buffer, pH 5.1 or 7.3. As MES has a pKA around 6.15 at 20 °C, it has decent buffer capacity at both pH 5.1 and 7.3. This means that measurements at the two different pH levels can be conducted with the same buffer, which facilitates comparison. For Aβ_40_ at pH 7.3, measurements were also conducted in the presence of 50 mM SDS surfactant. The samples were placed in a quartz cuvette with 4 mm path length, holding a total sample volume of 600 μL. Small aliquots of Hg(NO_3_)_2_ were titrated to the samples (less than 3% of total volume added) using stock solutions of 2, 3, and 10 mM Hg(NO_3_)_2_, respectively. The measured tyrosine fluorescence intensities were then plotted against the concentration of Hg ions. Dissociation constants (*K*_D_) for the Aβ·Hg complexes were calculated by fitting the data curves to Equation (1), the Morrison equation^[^ ^114^ ^]^

(1) $$I\;=\;I_{0}+\frac{I_{\infty }-I_{0}}{2\cdot \left[\text{Aβ}\right]}\cdot \left(\left(K_{D}+\left[\text{Hg}\right]+\left[\text{Aβ}\right]\right)-\sqrt{{\left(K_{D}+\left[\text{Hg}\right]+\left[\text{Aβ}\right]\right)}^{2}-4\cdot \left[\text{Hg}\right]\cdot \left[\text{Aβ}\right]}\right)$$

Here, *I*_0_ is the initial fluorescence intensity with no added Hg ions, *I*_∞_ is the steady‐state intensity at the end of the titration, [Hg] is the concentration of added Hg ions, *K*_D_ is the dissociation constant, and [Aβ] is the peptide concentration. This version of the equation assumes that the peptide has a single binding site for Hg ions. Adding a linear term for the fluorescence quenching effect of free Hg ions appeared unnecessary.^[^ ^48^ ^]^ No corrections were made for possible interactions between the buffer and the Hg ions. The derived dissociation constants should therefore be considered as apparent, i.e., $K_{D}^{\text{App}}$. However, the buffer effects are likely small, as MES is a Good buffer devised to have minimal interactions with metal ions and other cations.^[^ ^115^ ^]^ For each condition, three separate samples were prepared and measured, allowing mean $K_{D}^{\text{App}}$ values to be calculated.

##### NMR Spectroscopy

NMR spectroscopy measurements were performed on a 700 MHz Bruker Avance spectrometer equipped with a cryoprobe. Two‐dimensional ^1^H,^15^N‐HSQC spectra were recorded at 5 °C for 92 μM monomeric ^15^N‐labeled Aβ_40_ peptide in 20 mM MES buffer at pH 5.1, (90/10 H_2_O/D_2_O), before and after addition of 92 μM Hg(NO_3_)_2_. The Topspin v.3.6.2 software was used to process the NMR spectra, and the crosspeaks were assigned according to previously published data at both neutral pH^[^ ^116^, ^117^, ^118^ ^]^ and at acidic pH.^[^ ^119^ ^]^

##### CD Spectroscopy

CD spectra were recorded between 195 and 250 nm, with 0.5 nm step size, using a Chirascan CD instrument (Applied Photophysics Ltd., UK) operating at 20 °C. A quartz cuvette with 2 mm pathlength was used to hold 600 μL of either 10 μM Aβ_40_ peptide or 10 μM Aβ_4–40_ peptide in 20 mM phosphate buffer, pH 7.3. Both samples also contained 50 mM SDS surfactant. As this concentration is much higher than the critical micelle concentration (cmc) of SDS, which in pure water at 25 °C is 8.2 mM,^[^ ^120^ ^]^ the SDS forms micelles that constitute a simple but efficient membrane model.^[^ ^103^ ^]^ As the SDS micelle concentration is much higher than the Aβ concentration, there should be no more than one Aβ peptide in each micelle.

To both Aβ variants, i.e., Aβ_40_ and Aβ_4–40_, the dissolved mercury salt Hg(NO_3_)_2_ was added in steps of 16, 56, 156, and 256 μM. The recorded spectra were processed with the Chirascan Pro‐Data v.4.4.1 software (Applied Photophysics Ltd., UK), including smoothing with an eight points Savitzky‐Golay smoothing filter.

## Conflict of Interest

The authors declare no conflict of interest.

## Data Availability

The data that support the findings of this study are available from the corresponding author upon reasonable request.
